# ZNF165 Is Involved in the Regulation of Immune Microenvironment and Promoting the Proliferation and Migration of Hepatocellular Carcinoma by AhR/CYP1A1

**DOI:** 10.1155/2022/4446805

**Published:** 2022-06-01

**Authors:** Peng Liu, Yan Zhou, Xin Dong, Biao Zheng, Bo Liang, Rui Liang, Zhong Liu, Li Li, Peng Gong

**Affiliations:** ^1^Graduate School of Dalian Medical University, Dalian, Liaoning 116044, China; ^2^Department of General Surgery & Institute of Precision Diagnosis and Treatment of Gastrointestinal Tumors, Shenzhen University General Hospital & Shenzhen University Clinical Medical Academy, Xueyuan Road 1098, 518055 Shenzhen, China; ^3^Carson International Cancer Center & Guangdong Provincial Key Laboratory of Regional Immunity and Diseases, Shenzhen University Health Science Center, Xueyuan Road 1066, 518060 Shenzhen, China; ^4^Department of Obstetrics & Carson International Cancer Research Center, Shenzhen University General Hospital and Shenzhen University Clinical Medical Academy, Xueyuan Road 1098, 518055 Shenzhen, China; ^5^Department of Emergency Surgery, The Second Affiliated Hospital of Dalian Medical University, Dalian, 116011 Liaoning, China

## Abstract

The strong tumorigenic capacity and treatment resistance made hepatocellular carcinoma (HCC) a huge threat to public health. ZNF165, the kruppel family of zinc-finger-containing transcription factors, is expressed in HCC; however, its specific role in HCC and the molecular mechanism are yet to be elucidated. In this study, we observed that ZNF165 was overexpressed in liver cancer tissues and the immune microenvironment; higher ZNF165 expression was correlated with lower overall survival in liver cancer patients. The ZNF165 knockdown in Bel7402 cells revealed the impairment of the tryptophan/kynurenine/AhR/CYP1A1 axis. Moreover, the knockdown of CYP1A1 significantly inhibited the proliferation and migration of HCC cells, and ZNF165 promoted the transcriptional activity of AhR by facilitating the nuclear translocation of CYP1A1. In conclusion, the present study argued that ZNF165 was highly expressed in liver tissues and the immune microenvironment. ZNF165 promoted the proliferation and migration of HCC cells by activating the tryptophan/kynurenine/AhR/CYP1A1 axis and promoting the expression of CYP1A1.

## 1. Introduction

Hepatocellular carcinoma (HCC) is the third most common cause of cancer-related mortality [1]. The 5-year survival rate for the patients not undergoing therapy is less than 5% [2]. In China, HCC accounts for roughly half of all new cancer cases. Unfavorably, 50% of individuals with intermediate or advanced HCC suffered recurrence in two years after treatment, whereas the 5-year recurrence rate could reach as high as 74% [3, 4]. The strong tumorigenic capacity and treatment resistance of HCC cells are primarily responsible for recurrence.

ZNF165, one of the kruppel family of zinc-finger-containing transcription factors, is found in the testis. It is reported that a series of cancers such as gastrointestinal cancer [5] and urinary bladder transitional cell carcinoma [6] expressed ZNF165. Maxfield et al. evaluated the activity of ZNF165 in triple-negative breast cancer (TNBC) by a lot of research data and loss-of-function screening strategy [7]. Their findings revealed that ZNF165 significantly promoted the growth of TNBC tumors. In addition, ZNF165 associated with SMAD3 to modulate transcription of the transforming growth factor beta- (TGF-*β*-) dependent genes [8]. Although previous study suggested that ZNF165 is expressed in HCC tissues [5], the mechanism by which ZNF165 contributes to the tumor growth of HCC still needs to be elucidated.

In this study, ZNF165 expression was investigated in liver cancer tissues and matched with adjacent normal samples through bioinformatics and experimental analyses. For identifying downstream factors and signaling pathways, ZNF165 was knocked down in Bel7402 cells and array analysis was carried out for analyzing differentially expressed genes (DEGs). DEGs were applied for signaling enrichment annotation, and the tryptophan signaling pathway and its factor cytochrome P450 family 1 subfamily A member 1 (CYP1A1) were chosen. The functions of ZNF165 in the tryptophan signaling factors and metabolites were investigated. CYP1A1 knockdown and overexpression were achieved in Bel7402 and HCCLM3 cells, and the specific effects of CYP1A1 on cell proliferation, invasion, and drug resistance were investigated. Traditionally, aryl hydrocarbon receptor (AhR) could target the cytochrome P450 family 1 enzymes including CYP1A1 [9, 10]. Therefore, the regulation of AhR coordinated with ZNF165 was investigated. Finally, the relationship between ZNF165 and AhR, CYP1A1, or CYP1B1 was investigated. Our findings establish that ZNF165 expression is upregulated in HCC and contributes to oncogenic in HCC. Collectively, this study is aimed at investigating the specific effects and mechanism of ZNF165/AhR/CYP1A1 on liver cancer cell aggressiveness.

## 2. Materials and Methods

### 2.1. Clinical Sample Collection

A total of five liver cancer samples and five matched adjacent normal samples were collected from liver cancer patients and confirmed by postoperative pathology. All samples were taken from individuals having surgical resection at the First Affiliated Hospital of Dalian Medical University. The enrolled patients all signed an informed consent form. All clinical sampling was performed with the agreement of the Ethics Committee at the First Affiliated Hospital of Dalian Medical University (Approval No. YJ-KY-FB-2019-12). Tissues were promptly stored at -80°C following harvesting.

### 2.2. Immunohistochemical (IHC) Staining

Tissue samples were embedded in paraffin and cut into 4 *μ*m thick slices after being fixed in 4% paraformaldehyde. After dewaxing and hydrating, the tissue sections were permeabilized with 0.2% Triton X-100 (Sigma, St Louis, MO, USA) for 10 minutes at room temperature and incubated for 30 minutes with blocking solution. Then, samples were treated with anti-ZNF165 antibody (Novus Biologicals, USA; Code: H00007718-B01P) followed by a 30-minute incubation with poly-HRP antibody (Cell Signaling Technology, USA; Code: 7074). The slices were subsequently stained with a DAB staining kit (Beyotime; Code: P0203), and the nucleus was labelled with hematoxylin (0.2 mg/ml, Sigma-Aldrich). Finally, the slices were examined under a microscope.

### 2.3. Immunoblotting

After being incubated by indicated transfection and treatment, cells were washed with PBS and added to RIPA (PMSF) buffer. The detailed procedures were reported in our previous study [11]. In brief, the cell lysates were separated on SDS polyacrylamide gels and the proteins were transferred to the polyvinyl difluoride membrane. Subsequently, the membranes were blocked with 2.5% BSA and incubated with the primary antibodies: ZNF165 (OriGene, USA; Code: TA308554), AhR (Proteintech, USA; Code: 17840-1), Lamin A+C (Cell Signaling Technology, USA; Code: 4777), or GAPDH (Proteintech; Code: 104941-1-AP) overnight at 4°C. Next, the membranes were incubated for 1 hour at room temperature with proper secondary antibodies (HRP-labeled goat anti-rabbit IgG (Cell Signaling Technology; Code: 7074) and goat anti-mouse IgG (Cell Signaling Technology; Code: 7076)) and washed three times with 0.05% TBST for 5 minutes each time. The ECL chemiluminescence method was used to detect the signal.

### 2.4. Cell Lines

A hepatocellular carcinoma cell line Bel7402 was obtained from the Cell Bank of the Chinese Academy of Sciences (Shanghai, China). A highly metastatic hepatocellular carcinoma cell line HCCLM3 was obtained from Liver Cancer Institute (Zhongshan Hospital, Fudan University, Shanghai, China). In 5% CO_2_ environment at 37°C, Bel7402 cells were cultured in RPMI 1640 medium (Gibco; Code: 11875101), whereas HCCLM3 cells were cultured in DMEM/GlutaMax-1 (Gibco; Code: 11965092) supplemented with 10% FBS (Gibco; Code: 10099141) and 100 IU/ml penicillin/streptomycin (Gibco; Code: 15140122).

### 2.5. Cell Transduction

For downregulation of ZNF165, the shZNF165-1/2 vectors were used and the sh-CTR vector was employed as a negative control. For ZNF165 overexpression, the ZNF165-overexpressing plasmid (OE-ZNF165) was synthesized, and the OE-CTR plasmid was employed as a negative control. Similarly, for CYP1A1 knockdown, the shCYP1A1 vector was synthesized, and the sh-CTR vector was employed as a negative control. For CYP1A1 overexpression, the CYP1A1-overexpressing plasmid (OE-CYP1A1) was synthesized, and the OE-CTR plasmid was employed as a negative control. For AhR knockdown, the shAhR vector was synthesized and the sh-CTR vector was employed as a negative control. Following the manufacturer's instructions, these vectors were transfected successfully into target cells using Lipofectamine 3000 Reagent (Invitrogen).

### 2.6. qRT-PCR

To investigate the expression of ZNF165, AhR, CYP1A1, and CYP1B1 (a homologous protein of CYP1A1) in HCC cells, the qRT-PCR was performed and the primer sequences are summarized in Supplementary Table S1. Total RNA was isolated from digested and lysed cells using an RNA isolation kit (Tiangen Biotech, China) according to the manufacturer's instructions. Thereafter, 5 *μ*l of RNA was collected from each group and the RNA diluted with RNase-free ultrapure water. Invitrogen 2x SYBR Real-Time Mix and the MxPro System were exploited for reverse transcription. The 2−*ΔΔ*CT technique was used to calculate the expression levels.

### 2.7. ELISA

The levels of kynurenine and tryptophan in the supernatant were evaluated using corresponding ELISA kits (MyBioSource, MBS495082 and MBS495079, respectively). Cells were transduced and treated accordingly. The supernatant from the target cells was collected and centrifuged for 5 minutes at 1500 rpm. Subsequently, the level of kynurenine and tryptophan was measured using ELISA kits following the manufacturer's instructions.

### 2.8. Colony Formation

Following transduction and treatment, 1 × 10^5^ cells were plated in each of the six-well plates coated with 0.6% agarose. The cells were cultured on agarose for two weeks at 37°C in 5% CO_2_, and the resulting colonies were counted after staining with 0.1% crystal violet. Colonies with more than 50 cells were counted manually.

### 2.9. MTT Assay

Following transduction and treatment, 5 × 10^3^ cells were plated in each well of the 96-well plates. At each time point, 20 *μ*l MTT (5 mg/ml, Invitrogen) was added to each well followed by incubation in a humidified incubator for 4 hours. After draining the supernatant, the formazans were dissolved in DMSO. The optical density values were evaluated at 450 nm.

### 2.10. Transwell Assay Detecting Cell Migration

**Figure 1:**
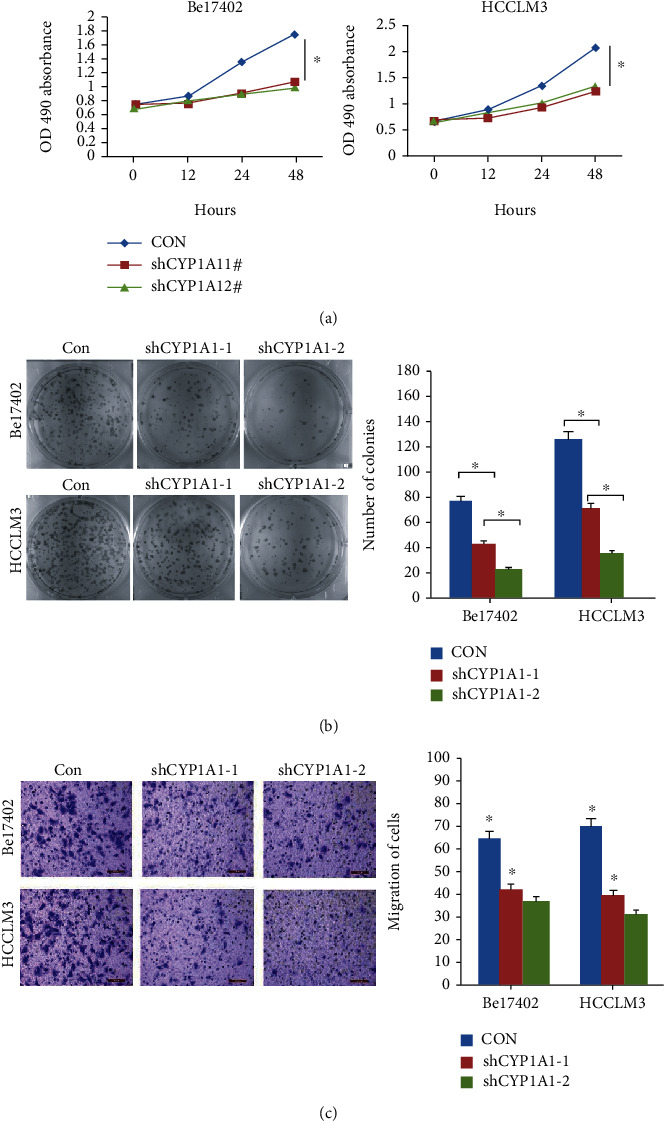
Specific effects of CYP1A1 knockdown and overexpression on liver cancer cell aggressiveness. (a) CYP1A1 knockdown significantly inhibited liver cancer cell viability. (b) CYP1A1 knockdown significantly inhibited liver cancer cell colony formation. (c) CYP1A1 knockdown remarkably suppressed liver cancer cell migration.

Transwell plates precoated with Matrigel in the top chambers were used for the invasion analysis. Transduced and/or treated cells were digested with 0.25% trypsin before being suspended in serum-free media and counted. Then, in the upper compartment, 200 *μ*l (5 × 10^5^ cells/ml) of cells was seeded, whereas 600 *μ*l of DMEM containing 10% FBS was added to the lower chamber. The top chamber was rinsed twice with PBS after 24 hours of incubation. A cotton swab was used to remove the cells on the top surface. The remaining cells after passing through the basement membrane were fixed in anhydrous methanol and stained with 0.1% crystal violet. Under an optical microscope, the number of membrane invaded cells was counted, and representative images acquired during the experiment were shown (Figure 1(c)).

### 2.11. Luciferase Reporter Assay

In order to elucidate the transcriptional activity of AhR, the luciferase reporter experiment was performed. Target cells (3 × 10^4^ cells/well) were plated in 24-well plates and grown for 24 hours. Lipofectamine 3000 Reagent (Invitrogen, L3000150) was used to transfect the appropriate plasmids of pGL3-AhR as well as 1.5 ng of the pRL-TK renilla plasmid (Promega). A Dual-Luciferase Reporter Assay Kit (Promega, E1910) was used to assess luciferase and renilla signals 48 hours after transfection, according to the manufacturer's instructions.

### 2.12. Immunofluorescent (IF) Staining

Cells were transduced and routinely sown on coverslips. The cells were fixed in 4% paraformaldehyde for 15 minutes on reaching 70% of confluence. After washing with PBS, the cells were incubated for 20 minutes with 0.1 ml Triton X-100 (0.5%) and then blocked with 10% bovine serum albumin for 20 minutes. The cells were then treated with anti-AhR for 8-10 hours at 4°C. After rinsing with PBS, the cells were incubated with proper fluorescein-conjugated secondary antibodies for 1 hour. Thereafter, 4,6-diamidino-2-phenylindole (DAPI, Sigma, D9542) was used for nuclear staining. Finally, a laser scanning confocal microscope (Leica TCS SP5x) was used to monitor the immunofluorescence.

### 2.13. Statistical Analysis

SPSS version 21.0 was used for all statistical analyses (SPSS Inc., New York, NY, USA). The comparisons between groups were assessed using the two-tailed paired Student's *t*-test. The Kaplan-Meier method was used to plot survival curves, and the log-rank test was applied to compare the survival curves. Cox regression models, both univariate and multivariate, were used to assess survival data. Statistical significance was defined by the *p* value less than 0.05.

## 3. Results

### 3.1. Expression of ZNF165 in Liver Cancer Tissues

**Figure 2:**
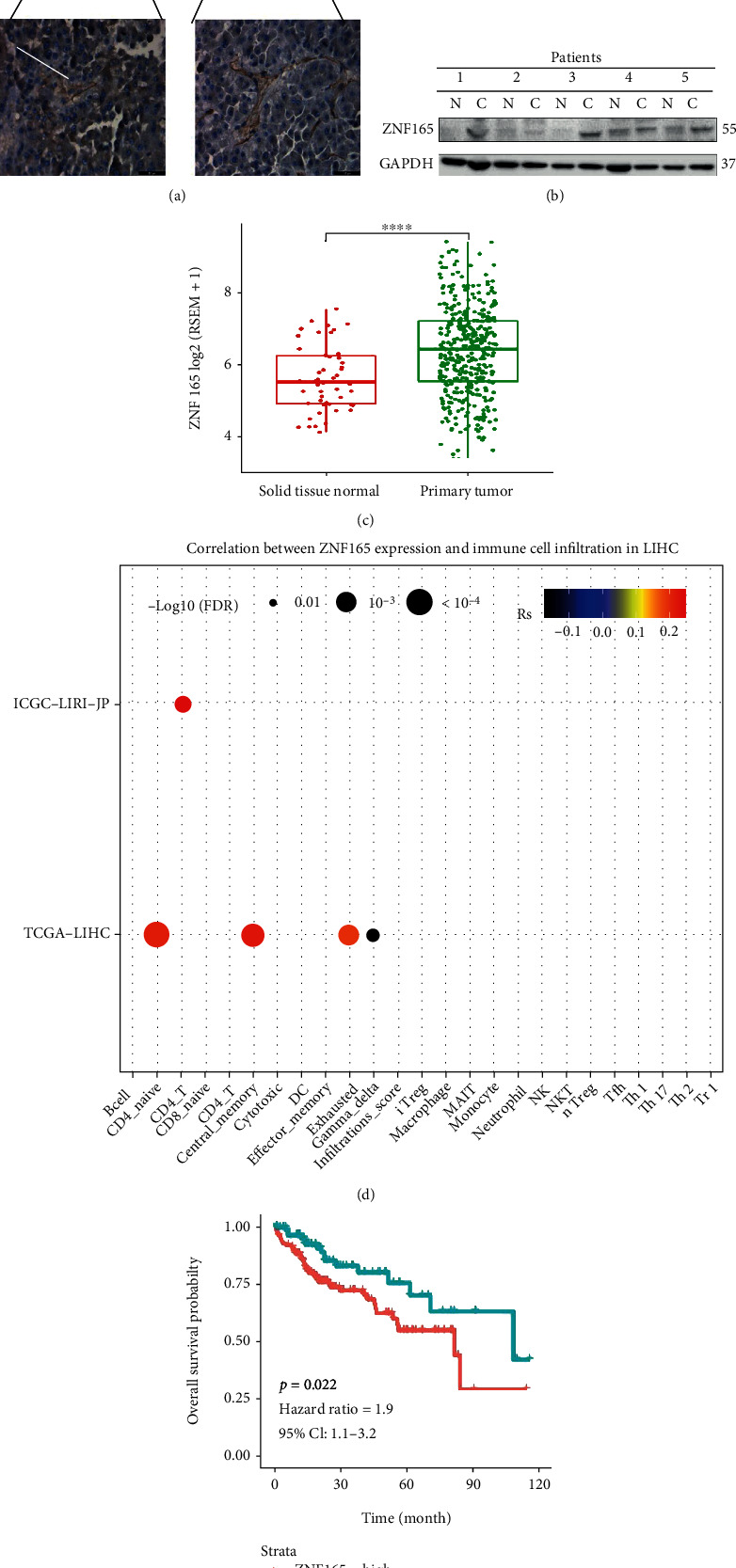
Expression of ZNF165 in liver cancer tissues. (a) The protein levels and distribution of ZNF165 in liver cancer tissues and liver normal tissues were detected by immunohistochemical (IHC) staining. (b) The protein levels of ZNF165 in liver cancer tissues and liver normal tissues were evaluated by immunoblotting. (c) Cases in TCGA and ICGC were divided into the high- and low-ZNF165 expression related to immune cell infiltration. (d) The mRNA expression of ZNF165 in liver cancer tissues and liver normal tissues was analyzed based on TCGA database. (e) Correlation between ZNF165 expression and the overall survival in liver cancer patients.

In this study, it was observed that the expression of ZNF165 was high in tumor tissues as compared to adjacent cancer tissues (Figure 2(a)). Consistent with the results of IHC, the western blot also revealed high expression of ZNF165 in the cancer tissues (Figure 2(b)). The above results were confirmed by the data from TCGA data bank. The data from TCGA data bank revealed that the mRNA expression of ZNF165 was significantly upregulated in liver cancer tissues as compared to paraneoplastic normal tissues (Figure 2(c)). In addition, we observed that ZNF165 was expressed in the immune microenvironment and its expression was positively correlated with CD4 naïve cells, central memory cells, exhausted cells, and gamma delta cells (Figure 2(d)). The high expression of ZNF165 impaired the survival of liver cancer patients and increased the death risk by 1.9-fold (Figure 2(e)). Moreover, the Cox regression analysis revealed that ZNF165 is an independent risk factor of survival (Table 1).

### 3.2. ZNF165 Regulates the Tryptophan Signaling by CYP1A1

**Figure 3:**
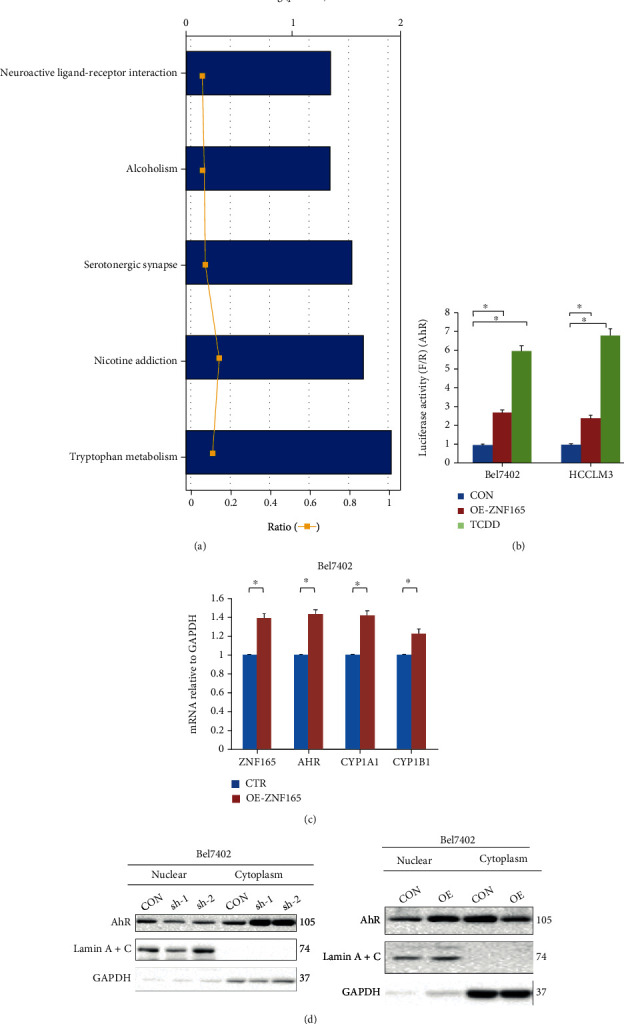
CYP1A1 in tryptophan signaling can be regulated by ZNF165. (a) ZNF165 knockdown was achieved in Bel7402 cells by transducing short hairpin RNA targeting ZNF165 (sh-ZNF165-1/2), and differentially expressed genes (DEGs) were analyzed using array assay. DEGs were applied for signaling pathway enrichment annotation. (b, c) ZNF165 knockdown or overexpression was achieved in Bel7402 and HCCLM3 cells by transducing sh-ZNF165-1/2 or ZNF165-overexpressing plasmid (OE-ZNF165), and the mRNA expression of the tryptophan pathway-related genes AhR, CYP1A1, and CYP1B1 was determined using qRT-PCR.

For investigating the mode of action of ZNF165 affecting liver cancer progression, factors and signaling pathways that might be modulated by ZNF165 were first analyzed. ZNF165 knockdown was acquired in Bel7402 cells by transducing sh-ZNF165-1/2, and differentially expressed genes (DEGs) were analyzed using the array. Herein, in this study, it was observed that genes were enriched in the tryptophan metabolism signaling pathway as compared to the wild-type cells (Figure 3(a)), suggesting that ZNF165 can regulate the tryptophan signaling pathway. In order to evaluate this observation, ZNF165 was downregulated or overexpressed in Bel7402 and HCCLM3 cells; and the tryptophan pathway-related genes AhR, CYP1A1, and CYP1B1 were evaluated at the RNA level. Furthermore, the downregulation of ZNF165 could significantly decrease the expression of AhR, CYP1A1, and CYP1B1 in liver cancer cells (Figure 3(b)). Finally, overexpression of ZNF165 significantly increased the transcription of AhR, CYP1A1, and CYP1B1 (Figure 3(c)).

### 3.3. ZNF165 Regulates AhR Transcriptional Activity

**Figure 4:**
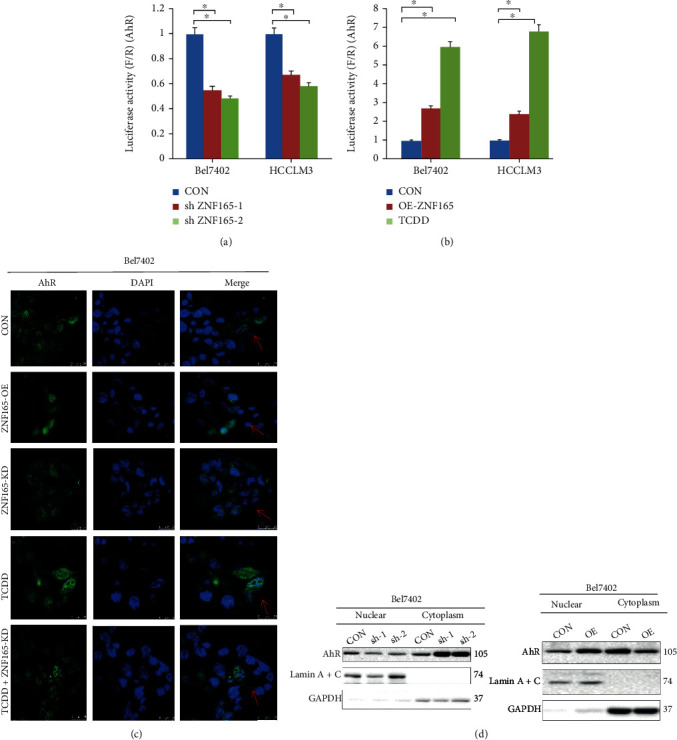
ZNF165 regulates AhR transcriptional activity. (a) ZNF165 overexpression significantly facilitated the luciferase activity of pGL3-AhR promoter, similar to TCDD a positive control of inducing AhR nuclear translocation. (b) The luciferase activity of the pGL3-AhR promoter was significantly inhibited by ZNF165 knockdown. (c) The overexpression of ZNF165 increased, while the knockdown of ZNF165 reduced AhR nuclear translocation. As a further confirmation, the nuclear and cytoplasm levels of AhR protein were examined in shZNF165-1/2- or OE-ZNF165-transduced cells. (d) After ZNF165 overexpression, AhR levels were significantly increased in the nucleus and decreased in the cytoplasm. After knocking down of ZNF165, AhR levels were significantly decreased in the nucleus and increased in the cytoplasm.

In order to evaluate the mechanism of how ZNF165 regulates the transcription of AhR, the pGL3-AhR promoter-luciferase reporter plasmid was constructed and cotransduced into Bel7402 or HCCLM3 cells and the luciferase activity was determined. Luciferase analysis revealed that downregulation of ZNF165 significantly decreased the luciferase activity of AhR (Figure 4(a)). In addition, we observed that overexpression of ZNF165 (OE-ZNF165) and treating the cells with tetrachlorodibenzo-p-dioxin (TCDD) significantly increased the luciferase activity as compared to control (CON) (Figure 4(b)). The aforementioned finding was further supported by immunofluorescent staining and immunoblotting. The immunofluorescent staining revealed that overexpression of ZNF165 promotes the AhR to enter the nucleus (Figure 4(c)). Additionally, Bel7402 and HCCLM3 cells were treated with sham (CON) or a short hairpin RNA of ZNF165 (sh-1 and sh-2) or the plasmid of ZNF165. The finding revealed that downregulation of ZNF165 by the short hairpin RNA resulted in increased accumulation of AhR in the cytoplasm. However, overexpression of ZNF165 reduced the cytoplasmic level of AhR (Figure 4(d)).

### 3.4. ZNF165 Regulates the Secretion of Kynurenine and Tryptophan

**Figure 5:**
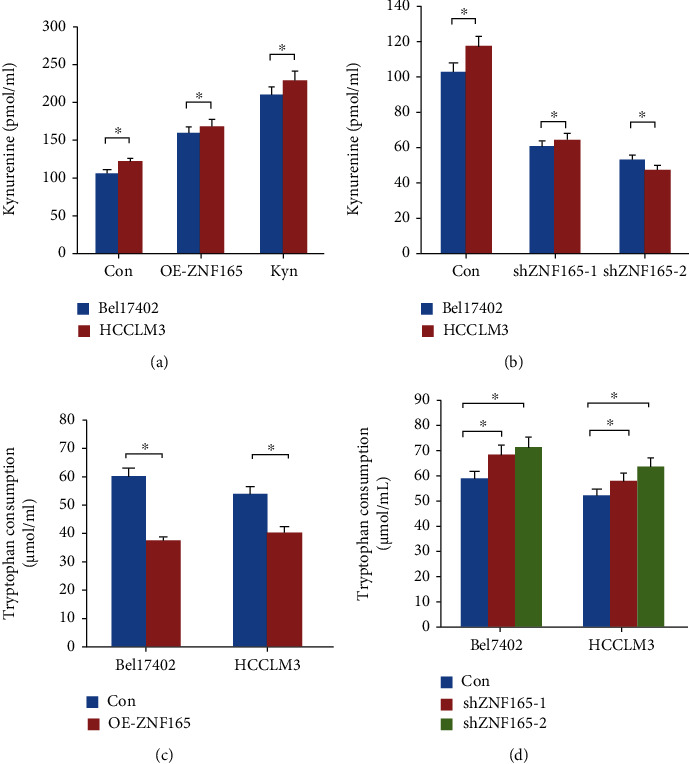
ZNF165 affects tryptophan metabolites. (a, b) Significantly decreased kynurenine content in the supernatant of cells knocked down with ZNF165 was detected, whereas increased kynurenine content was detected in the supernatant of cells overexpressing ZNF165. (c, d) A significant increase in tryptophan content in the supernatant of cells with knocked down ZNF165 was detected, whereas increased tryptophan content was detected in the supernatant of cells overexpressing ZNF165.

In order to evaluate whether ZNF165 is involved in the metabolism of kynurenine and tryptophan, we overexpressed ZNF165 in liver cancer cells. We observed that overexpression of ZNF165 significantly increased the accumulation of kynurenine in the supernatant of liver carcinoma cells (Figure 5(a)). A significant decrease in kynurenine was observed when ZNF165 was downregulated (Figure 5(b)). Moreover, the overexpression of ZNF165 decreased (Figure 5(c)) while knockdown of ZNF165 increased the level of tryptophan in the supernatant (Figure 5(d)), suggesting that ZNF165 might inhibit the process of kynurenine production by tryptophan.

### 3.5. ZNF165 Promotes the Proliferation and Migration of Liver Cancer Cells by CYP1A1

In order to investigate whether ZNF165 regulates the cell phenotypes by CYP1A1, we knocked down CYP1A1 and evaluated the proliferation and migration of the liver cancer cells. We observed that CYP1A1 downregulation significantly inhibited the viability of Bel7402 and HCCLM3 cells (Figure 1(a)). In addition, downregulation of CYP1A1 could impair its ability to form clones in liver cancer cells (Figure 1(b)). Furthermore, the Transwell analysis revealed that the downregulation of CYP1A1 significantly inhibits the migration of Bel7402 cells and HCCLM3 cells (Figure 1(c)).

## 4. Discussion

ZNF165 is a member of the C2H2 zinc finger protein family [12]. Previous study suggests that ZNF165 is specifically expressed in the testis and tumor tissues and involved in the development of various malignant tumors, such as breast cancer [8], urinary bladder cancer [6], and hepatocellular carcinoma patients [3, 9]. Previously, it was reported that ZNF165 is a pro-oncogene and promotes the proliferation and migration of breast cancer cells by upregulating the TGF-*β* signaling pathway [8].

In the present study, a higher expression of ZNF165 was observed in liver cancer tissues which was correlated with lower overall survival and progression-free survival. After knockdown of ZNF165 in Bel7402 cells, the tryptophan signaling pathway factors, particularly CYP1A1, as well as tryptophan and kynurenine, two metabolites of tryptophan signaling pathway, were downregulated. In addition, we observed that ZNF165 promotes the transcriptional activity of AhR by facilitating the nuclear translocation of AhR. In AhR knockdown cells, the overexpression of ZNF165 did not increase CYP1A1 expression. Similarly, after adding CH-223191, an inhibitor of AhR, the CYP1A1 expression was not increased by the overexpression of ZNF165, indicating that ZNF165 affects CYP1A1 expression through AhR.

Previously, frequent expression of ZNF165 has been reported in various cells [3, 5, 6, 8, 9]. The ZNF165, by directly inactivating the expression of negative feedback pathway regulators: SMURF2 [13], SMAD7 [14], and PMEPA1 [15], promotes the unrestrained activation of transforming growth factor *β* (TGF*β*) signaling, which is required for the survival of triple-negative breast cancer cells *in vitro* and *in vivo* [7, 8]. In this study, possible downstream pathways and factors that might be affected by ZNF165 were first analyzed; the tryptophan signaling pathway factors particularly CYP1A1 were downregulated after knocking down ZNF165 in HCC cells. The tryptophan-kynurenine-aryl hydrocarbon receptor pathway is involved in physiological immune suppression and also plays a vital role in acquired and intrinsic immunotherapy resistance [16]. In this study, downregulation of ZNF165 significantly decreases the expression of CYP1A1, as well as the level of tryptophan signaling metabolites: tryptophan and kynurenine. A previous study reported linkage between CYP1A1 polymorphisms and the risk of developing HCC [16]. Being the most active enzyme in converting procarcinogens into active compounds, CYP1A1 promotes the formation of DNA adducts [17, 18] and bioactivation of exogenous procarcinogens of HCC [19]. Another study demonstrated that GNMT exerted its antitumor activities on hepatocarcinogenesis partially through binding to the CYP1A1 promoter region and inhibiting CYP1A1 expression [20]. The current study first investigated the specific cellular functions of CYP1A1 knockdown and overexpression in HCC cell aggressiveness. Consistent with previous study conducted by [5], CYP1A1 overexpression performed oncogenic functions by promoting HCC cell proliferation, invasion, and mobility. After knocking down CYP1A1, the malignant behaviors of HCC cells have been remarkably eliminated. Considering our earlier experimental results and previous findings, CYP1A1 might mediate the functions of ZNF165 on HCC cells, and knocking down CYP1A1 might represent a promising strategy for HCC treatment regimens.

Canonically, AhR mainly targets the cytochrome P450 family 1 enzymes, including CYP1A1 [21, 22]. When a ligand binds AhR, it translocates to the nucleus, where it induces transcription of CYP1A1 followed by an increase in CYP1A1 enzymatic activity [23, 24]. Considering that ZNF165 overexpression upregulated CYP1A1 expression, the current study investigated whether ZNF165 could affect AhR transcriptional activity and expression. As expected, while cotransducing OE-ZNF165 and pGL3-AhR promoter-reporter plasmid, the luciferase activity of the pGL3-AhR was significantly enhanced, which was similar to the results induced by TCDD, an inducer of AhR nuclear translocation. In contrast, shZNF165 transduction significantly eliminated the luciferase activity of the pGL3-AhR, suggesting that ZNF165 might promote the transcriptional activity of AhR. For further verification, the dynamic effects of ZNF165 and AhR on AhR, CYP1A1, and CYP1B1 were also investigated. Consistent with earlier speculation, after knocking down AhR, ZNF165 overexpression caused no upregulation of CYP1A1 expression; moreover, after adding the AhR inhibitor CH-223191, ZNF165 overexpression also failed to upregulate CYP1A1 expression. These findings indicated that ZNF165 upregulated CYP1A1 by promoting the transcriptional activity of AhR. Consistently, the present study revealed the higher ZNF165 levels in liver cancer samples both in TCGA data and experimental results. Compared with other related studies, we found for the first time that ZNF165 regulates the tryptophan pathway to effect cell growth and migration of HCC. More importantly, higher expression of ZNF165 was significantly found associated with lower overall survival and progression-free survival in liver cancer patients, suggesting that ZNF165 might play a role in liver cancer progression. Although we found that ZNF165, as a new oncogene, promoted the development of HCC and was predicted to have tumor immunomodulatory function, the specific regulatory mechanism should still be further studied. We will further explore its immune regulation mechanism in the follow-up studies. We need a large number of clinical samples to confirm the clinical significance and value of ZNF165.

**Figure 6:**
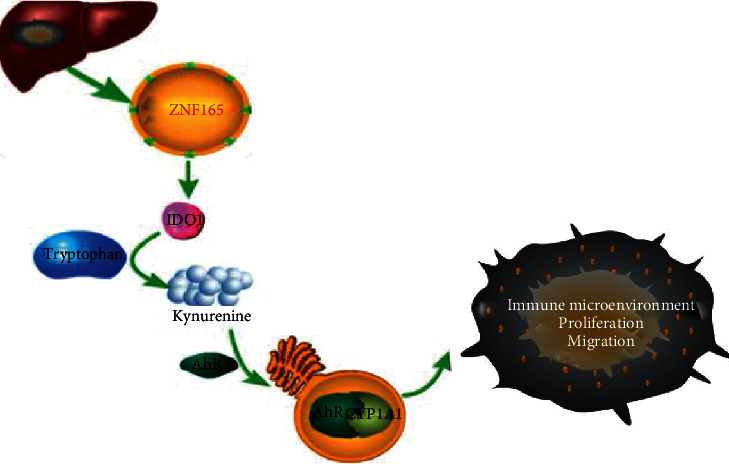
A schematic diagram showing the mechanism of ZNF165 modulating CYP1A1 through AhR, therefore affecting liver cancer cell aggressiveness.

In conclusion, ZNF165 serves as an oncogenic factor in HCC by regulating the immune microenvironment and promoting HCC cell proliferation and invasion. This possibly may be accomplished by upregulation of CYP1A1 expression via enhancing AhR transcriptional activity (Figure 6).

## Figures and Tables

**Table 1:** The univariate and multivariate Cox regression.

Variables	Univariate analysis			Multivariate analysis		
	HR	95%CI	p value	HR	95%CI	p value
ZNF165	2.519	1.486-4.268	0.001	2.000	1.113-3.592	0.020
Sex	1.426	0.569-3.572	0.449			
Grade	1.975	1.135-3.437	0.016	0.192	0.818-2.729	1.494
Age	0.950	0.547-1.649	0.855			
T stage	2.053	1.369-3.079	0.001	2.593	0.596-11.282	0.204
N stage	3.075	0.415-22.778	0.272			
M stage	41.166	3.733-453.988	0.002	21.085	1.043-426.125	0.047
TNM stage	2.148	1.392-3.313	0.001	0.703	0.153-3.230	0.651
KI67	1.509	0.797-2.854	0.206			
P53	1.277	0.745-2.190	0.374			
AFP	0.913	0.535-1.556	0.737			
CD34	0.874	0.509-1.503	0.627			

## Data Availability

The datasets are available from the corresponding author on reasonable request.

## Supplementary Materials

Supplementary Materials Supplementary Table 1: the sequences used in qRT-PCR.
