# Supplementary material for: ZNF165 Is Involved in the Regulation of Immune Microenvironment and Promoting the Proliferation and Migration of Hepatocellular Carcinoma by AhR/CYP1A1
Source: J Immunol Res. 2022 Jun 1;2022:4446805. doi: 10.1155/2022/4446805 (PMC9177304; doi:10.1155/2022/4446805)
Supplement: Supplementary Materials — Supplementary Table 1: the sequences used in qRT-PCR. [file 4446805.f1.pdf]

Supplementary Table 1. The sequences used in qRT-PCR.

Gene	Sequence
ZNF165	F: ACCAAGGCCCATTTTGATTCA<br>R: CTCTGAGACTCCCCTGATTCTT
AhR	F: CAAATCCTTCCAAGCGGCATA<br>R: CGCTGAGCCTAAGAACTGAAAG
CYP1A1	F: CTATCTGGGCTGTGG GCAA<br>R: CTGGCTCAAGCACAACTTGG
CYP1B1	F: AACCGCAACTTCAGCAACTT<br>R: GAGGATAAAGGCGTCCATCA
GAPDH	F: TGCACCACCAACTGCTTAG<br>R: GATGCAGGGATGATGTTC
